# Book Reviews

**Published:** 1901-04

**Authors:** 


### Book Reviews.

A System of Practical Therapeutics. By Eminent American and Foreign Authorities. Edited by Hobart Amory Hare, M. D., Professor of Therapeutics, Jefferson Medical College; Physician to Jefferson College Hospital, etc., Philadelphia. New (2d) edition, thoroughly revised. In three 8vo volumes, containing 2593 pages, with 427 engravings and 26 full-page colored plates. Philadelphia and New York: Lea Brothers & Co. 1901. [Price per volume, cloth, $5.00 net; leather, $6.00, net; half morocco, $7.00, net.]

The first and second volumes of this important system are already issued, and the third is to follow at a short interval of time. This second edition makes the work comparatively a new treatise, because nearly one-half of the articles are entirely new, while the others have been very largely rewritten. Moreover, the revision has been so carefully done that scarcely a paragraph fails to mark the handiwork of the reviser's pen. The following summary of Volume I. will prove interesting:

General therapeutic considerations, by Horatio C. Wood; Prescription-writing and the combination of drugs, by Joseph P. Remington; General sanitation, by Henry B. Baker; Nutrition and foods, including the treatment of obesity and leanness, by I. Burney Yeo; General exercise, by Edward Mussey Hartwell; The rest-cure for neurasthenia and hysteria, by John K. Mitchell; Electro-therapeutics, by A. D. Rockwell; Hydrotherapy, by Simon Baruch; Climate, by S. Edwin Solly; Mineral waters and their medicinal uses, by James K. Crook (new); Massage and swedish movements, by Robert E. Moore (new); Disinfection, by W. M. L. Coplin (new); Diseases of the thyroid and thymus glands, including myxedema, cretinism, Graves's disease, and obesity, by S. J. Meltzer; Chronic articular rheumatism, rheumatoid arthritis and gout, by James Stewart (new); Treatment of diabetes mellitus, by James Tyson (new); Diseases of the blood, by Ralph Stockman (new); The present treatment of syphilis, by Edward Martin; The treatment of tuberculosis, by Lawrence F. Flick (new); Scrofulosis, by Walter Chrystie; Scurvy, or scorbutus, by Charles Edward Banks (new).

It will be observed that each author has been carefully chosen, and special fitness for the subject written upon has been the paramount object in view.

The second volume presents authors and subjects as follows:

Typhoid fever, by H. A. Hare (new); Malarial fevers, by James M. Anders; Smallpox, by William M. Welsh; Varicella, rubeola, rubella and scarlatina, by J. P. Crozer Griffith (new); Yellow fever, by D. T. Laine (new); Dengue, by W. J. McLaughlin (new); Acute tonsillitis and influenza, acute articular rheumatism, by Frederick A. Packard (new); Diphtheria, by Floyd M. Crandall (new); Spasmodic croup and rickets, by Floyd M. Crandall (new); Diseases of the mucous membrane of the mouth, and mumps, by Floyd M. Crandall (new); Pneumonia, croupous and catarrhal, by H. A. Hare (new); Asthma, bronchitis and whooping cough, by Norman Bridge; Acute and chronic organic diseases of the heart, by W. H. Thomson; Diseases of the bloodvessels, by Frederick C. Shattuck; Nervous diseases of the heart, by Sir Lauder Brunton; Diseases of the stomach, by Thomas G. Ashton; Diseases of the liver, gall-bladder, hepatic duct and spleen, by John H. Musser (new); Diarrheal diseases and dysentery, by W. W. Johnston; The intestinal parasites, by H. A. Hare; Diseases of the kidneys, by N. S. Davis, Jr.; Headaches and neuralgia, by Wharton Sinkler; The drug habits, by F. X. Dercum; The disorders of sleep, by Hugh T. Patrick; Locomotor ataxia, acute infantile spinal paralysis, myelitis, and amyotrophic lateral sclerosis, by M. Allen Starr; Apoplexy, brain tumor, spinal tumor, meningitis, cerebritis, and neuritis, by Charles K. Mills; Spasmodic affections of the nervous system, by Joseph Collins; The medical treatment of insanity, by H. M. Bannister; Hospital insanity, by Edward N. Brush; The modern treatment of diseases of the skin, by Edward W. Stelwagon.

With such an array of talent, general and special, it is little wonder that the system should obtain a completeness rarely equaled, and that it should be authoritative in its teachings, accepted as such and so recommended by editors and instructors. It is, moreover, a work of almost indispensable necessity to the general practitioner of medicine, giving the latest information needed at the bedside in everyday practice.

---

THE CARE OF THE CONSUMPTIVE. A consideration of the scientific use of natural therapeutic agencies in the prevention and cure of consumption; together with a chapter on Colorado as a Resort for Invalids. By CHARLES FOX GARDINER, M. D., Member of the American Climatological Association. Duodecimo, pp. 182. New York and London: G. P. Putnam's Sons. 1900.

The purpose of this book, according to the author's statement, is to give practical rules for the consumptive regarding the use of fresh air, sunlight, food, rest and exercise; in other words, to instruct those afflicted with consumption how to apply nature's remedies to the best advantage.

In the course of eleven chapters the author treats of these subjects in a most intelligent and simple way. In these days when the state is interesting itself in the care of incipient consumptives in sanitoria specially located and chosen for that purpose, such a book as this cannot fail to be advantageous, by disseminating among the laity an intelligent understanding of the subjects under consideration. For instance, the chapter on living out of doors should be carefully studied, and especially to be commended is that portion of it relating to tent life. House hygiene is another chapter that appeals to the intelligent reader and, though short, it contains much common sense information. We commend the book most heartily to every person interested in the subject.

---

Obstetric Clinic. By Denslow Lewis, Ph. C., M. D., Professor of Gynecology in the Chicago Polyclinic; President of the Attending Staff of Cook County Hospital, Chicago. A series of thirty-nine clinical lectures on practical obstetrics delivered to Students and Practioners in Cook County Hospital, Chicago. Octavo, pp. 640. Chicago: E. H. Colegrove, 65 Randolph Street. [Price, $3.00.]

It is difficult to understand why this book has been published, except from the viewpoint of an advertisement, for it certainly does not contribute anything new to the literature of medicine.

The publication in book form of stenographic reports of clinical lectures is of doubtful propriety in general, and it seems to us in these days of overproduction of medical literature this book in particular may be spared without detriment.

One of the vagaries of the book is that the author's name appears at the head of each lecture, and we are as often reminded that each one is "stenographically reported by Bertha Barnet." Beginning with the nineteenth lecture the author adds "professor" to his name and title and so continues to the end. The book is printed on a poor quality of paper, but an attempt has been made to bind it rather handsomely, if not substantially.

STUDENTS' EDITION—A PRACTICAL TREATISE OF MATERIA MEDICA AND THERAPEUTICS, with Special Reference to the Clinical Application of Drugs. By JOHN V. SHOEMAKER, M. D., LL.D., Professor of Materia Medica, Pharmacology, Therapeutics and Clinical Medicine, and Clinical Professor of Diseases of the Skin in the Medico-Chirurgical College of Philadelphia; Physician to the Medico-Chirurgical Hospital. Fifth edition. Thoroughly revised. Pages vii.—770. Philadelphia: F. A. Davis Co. 1901. [Extra cloth, $4.00 net; sheep, $4.75 net.]

Experience in teaching develops the necessities of the class-room, the clinic and the laboratory. The author of this work has found from teaching a vast number of students, what they most need as a text-book in this department of medicine. This experience has led him to make a change in issuing the fifth edition of his well and favorably-known treatise. It now appears as two independent issues,—one for students, and the other for practitioners of medicine.

The first, known as the students' edition, is before us, and we cannot fail to commend the new departure in the highest terms. In the students' edition, the author has included only these drugs and preparations official in the pharmacopoeias of the United States and Great Britain. This is an excellent plan, as it is highly important not to confuse the student with too much material for him to assimilate. He should first become familiar with official remedies; this will absorb all the time he will find available during student life.

The author has taken pains to make his revision complete, so that it represents the immediate present in all the essentials of materia medica and therapeutics. It is one of the most practical text-books in this department of medicine.

---

A TEXT-BOOK OF PHARMACOLOGY AND THERAPEUTICS, OR THE ACTION OF DRUGS IN HEALTH AND DISEASE. For the use of Students and Practitioners of Medicine. By ARTHUR R. CUSHNY, M. A., M. D., Aberd., Professor of Materia Medica and Therapeutics in the University of Michigan Medical Department, Ann Arbor. New second edition. In one 8vo volume of 732 pages, with 47 engravings. Philadelphia and New York: Lea Brothers & Co. 1901. [Cloth, $3.75 net.]

The author of this work has a right to feel flattered by the reception given his first edition, which became exhausted in little more than a year. Starting out upon new lines as it did, some trepidation might have been felt as to its success, but now, with the profession practically in accord with the author, no further apprehension need be felt, and this edition is sure to meet the fate of the first—namely, early exhaustion.

Cushny aims to explain the reasons for drug action, and we think he has well succeeded in doing so, though this is one of the most difficult problems in therapeutics. The book is valuable, not alone for what it tells of accepted and acceptable drugs, but for warning against the unfounded claims of remedies that are untrustworthy. In a notice of the first edition (November, 1899,) we pointed out the fact that remedies which fail to withstand a rigid clinical test must stand aside in the treatment of disease. Cushny applies this test with scientific precision throughout his book.

The general plan of the treatise is unchanged, though it is kept abreast of the latest advances. We feel justified in the opinion first expressed of this excellent text-book, and are glad of the opportunity to reaffirm it with additional commendation.

